# Anoikis Resistance: An Essential Prerequisite for Tumor Metastasis

**DOI:** 10.1155/2012/306879

**Published:** 2012-02-23

**Authors:** Yong-Nyun Kim, Kyung Hee Koo, Jee Young Sung, Un-Jung Yun, Hyeryeong Kim

**Affiliations:** Pediatric Oncology Branch, National Cancer Center, 323 Ilsan-Ro, Ilsandong-Gu, Gyeonggi-Do, Goyang-Si 410-769, Republic of Korea

## Abstract

Metastasis is a multistep process including dissociation of cancer cells from primary sites, survival in the vascular system, and proliferation in distant target organs. As a barrier to metastasis, cells normally undergo an apoptotic process known as “anoikis,” a form of cell death due to loss of contact with the extracellular matrix or neighboring cells. Cancer cells acquire anoikis resistance to survive after detachment from the primary sites and travel through the circulatory and lymphatic systems to disseminate throughout the body. Because recent technological advances enable us to detect rare circulating tumor cells, which are anoikis resistant, currently, anoikis resistance becomes a hot topic in cancer research. Detailed molecular and functional analyses of anoikis resistant cells may provide insight into the biology of cancer metastasis and identify novel therapeutic targets for prevention of cancer dissemination. This paper comprehensively describes recent investigations of the molecular and cellular mechanisms underlying anoikis and anoikis resistance in relation to intrinsic and extrinsic death signaling, epithelial-mesenchymal transition, growth factor receptors, energy metabolism, reactive oxygen species, membrane microdomains, and lipid rafts.

## 1. Introduction

Development, differentiation, and homeostasis are controlled by cell-cell interactions, cell-extracellular matrix (ECM) interactions, and soluble cues (hormones, cytokines, and growth factors) [1, 2]. Cell adhesion to ECM occurs through interactions between specific integrin receptors and ECM counterparts. These interactions cause the transduction of many different signals that regulate cellular functions, such as gene expression, differentiation, proliferation, and motility. Importantly, an appropriate adhesion to ECM components determines whether a cell is in the correct location and thus regulates cell survival and cell death. In 1994, Frisch and Francis noticed that loss of matrix attachment of epithelial cells resulted in apoptosis [3]. They referred to this form of programmed cell death that occurs upon detachment from the appropriate ECM as anoikis [4–6]. Because anoikis prevents detached epithelial cells from colonizing elsewhere, thereby inhibiting dysplastic cell growth or attachment to an inappropriate matrix, anoikis is a physiologically relevant process for tissue homeostasis and development. Dysregulation of anoikis, such as anoikis resistance, is a critical mechanism in tumor metastasis. Epithelial cancers initially arise as an organ-confined lesion, but eventually spread to distinct organs through the bloodstream, generating metastatic lesions that are responsible for most cancer-related lethality. The tumor cells that acquire anoikis resistance can survive after detachment from their primary site and while traveling through the vascular system until they colonize the distal organ [4–7]. In addition, anoikis resistance is also important for the peritoneal dissemination of gastric and ovarian cancer cells [6, 8]. This paper will focus on the current understanding of cellular and molecular mechanisms of anoikis resistance.

## 2. Adhesion and Cell Survival

For survival and proliferation, normal epithelial cells require adhesion to specific ECM components through cell surface receptors known as integrins. Integrins are heterodimers consisting of *α*- and *β*-subunits. There are at least 24 distinct integrin heterodimers assembled by the combination of 18 *α*-subunits and 8 *β*-subunits. Because specific integrin heterodimers preferentially bind to distinct ECM components, the repertoire of integrins on the cell surface guides where the cell adheres or migrates. Integrin expression patterns vary between normal tissue and tumors [1, 6, 9]. Although integrins **α*v*β**1, **α**5**β**1, and **α*v*β**6 are usually expressed at low levels, they are highly upregulated in some tumors, whereas some integrins, such as **α**2**β**1, are decreased in tumor cells. Integrin ligation regulates not only cell adhesion and migration but also cell survival. Ligated integrins transduce survival signals, whereas unligated integrins can promote a proapoptotic cascade, thereby preventing cells from surviving in an inappropriate environment [2].

Integrins activate multiple signaling pathways that regulate cell motility and survival through interactions with cytoplasmic kinases, small G-proteins, and scaffolding proteins. Integrin ligation activates FAK, a nonreceptor tyrosine kinase, and activated FAK phosphorylates itself and other cellular proteins. FAK autophosphorylation at Y397 provides a binding site for SH2 domain-containing proteins such as Src family kinases and the p85 subunit of PI3K, which is important for integrin-mediated cell growth and migration [2, 6]. Integrin ligation activates the NF-*κ*B and PI3K/Akt pathways and upregulates prosurvival proteins, Bcl-2 and FLIP, thereby enhancing cell survival [10]. Cross-talk between growth factor receptors and integrins activates Raf, which also promotes cell survival [6]. Although cell adhesion is critical for cell growth in normal tissues, adhesion-dependent control of cell growth is dysregulated in tumor cells, as anchorage-independent growth is prominently implicated in malignant transformation. 

Recently, CUB domain-containing protein 1 (CDCP1), a transmembrane glycoprotein, has been linked to a noble pathway of anoikis resistance independent of Ras-MAPK and PI3K-Akt pathways in lung cancer and gastric cancer cell lines [11, 12]. Cell detachment induces CDCP1 phosphorylation by Src family kinases (SFKs) including Src, Fyn, and Yes. Upon SFK-mediated tyrosine phosphorylation of CDCP1, PKC*δ* forms a complex with CDCP1 and SFKs, is phosphorylated by SFKs and activated, leading to anoikis resistance [12]. Interestingly, CDCP1 phosphorylation by SFKs further activates SFKs and enhances metastasis in melanoma, indicating a “feed-forward loop” to maintain elevated activity of SFKs during tumor progression although the cause of the initial activation of SFKs is not clear [11, 13].

Resistance to detachment-induced cell death, or anoikis resistance, is emerging as a hallmark of metastatic malignancies, because it can ensure anchorage-independent growth and survival during tumor dissemination [4–6]. Numerous studies have suggested that stimulation of prosurvival signals and suppression of death signals are involved in anoikis resistance.

## 3. Prosurvival Signals and Apoptotic Signals in Anoikis Resistance

**Figure 1:**
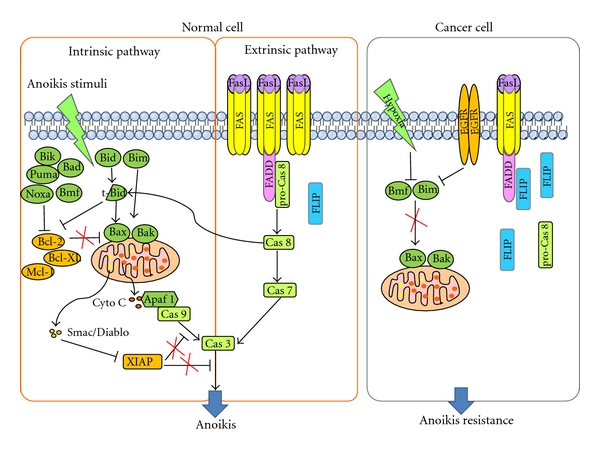
Schematic representation of intrinsic and exrtinsic pathways of anoikis. When cells are detached from ECM, normal cells induce anoikis through both intrinsic and exrtinsic pathways. Upon cell detachment, FAS and FasL are upregulated and FLIP is downregulated, leading to activation of Caspase 8, followed by activation of caspase-7 and caspase-3. Loss of cell adhesion also increases and activates proapoptotic Bcl-2 proteins (Bik, Puma, Bad, Noxa, Bmf, Bid, Bim, Bax, and Bak), which inactivate antiapoptotic Bcl-2 proteins (Bcl-2, Bcl-xL, Mcl-1), and thus causing mitochondria membrane permeabilization through Bax/Bak oligomerization. Released cytochrome c from mitochondria activates caspase-9, subsequently caspase-3. Smac/DIABLO is released and inhibits XIAP, an inhibitor of apoptosis, leading to caspase-3 activation. Activaiton of these pathways leads to anoikis. However, increased FLIP expression in cancer cells inhibits extrinsic pathway and oncogene expression such as EGFR and hypoxia downregulate Bmf and Bim, resulting in inhibition of mitochondrial pathway in suspended cells. Accordingly, cancer cells acquire anoikis resistance.

The role of the death receptor and the mitochondrial pathway in anoikis and anoikis resistance has been reviewed recently [5]. The death receptor (extrinsic) pathway is initiated by ligation of members of the TNF receptor superfamily, such as Fas and TNF-R1, causing the formation of the death-inducing signaling complex (DISC) as described in the Figure 1. Upon DISC formation, caspase-8 (FLICE) is activated and released into the cytoplasm where it cleaves caspase-3 and caspase-7. These activated effector caspases cleave cellular substrates, culminating cell death. FLIP (FLICE-inhibitory protein) has a higher affinity for the DISC than caspase-8 and is preferentially recruited to the DISC, thereby inhibiting caspase-8 recruitment and activation [5, 14, 15]. Anoikis employs this extrinsic pathway, at least partially. For example, matrix attachment protects cells from Fas-induced apoptosis, whereas matrix detachment sensitizes cells to Fas-mediated apoptosis. After cell detachment, FasL and Fas receptor are upregulated but FLIP, an endogenous antagonist of caspase-8, is downregulated, which leads to caspase-8 activation in human umbilical vein cells (HUVECs) [16, 17]. In addition, unligated integrin recruits caspase-8 to the membrane, where it becomes activated in a death receptor-independent manner, whereas integrin ligation disrupts this integrin-caspase complex and increases survival [18]. Caspase-8 activation triggers anoikis but FLIP overexpression, which inhibits caspase-8 activation and blocks anoikis in keratinocytes and HUVECs [16, 17]. Malignant cells avoid anoikis by overexpressing FLIP [5]. Anisomycin can sensitize cells to anoikis by decreasing FLIP protein levels and inhibits distal tumor formation in a mouse model of prostate cancer metastasis [19].

 In the mitochondrial (intrinsic) pathway, proteins of the Bcl-2 family are key players and include anti-apoptotic proteins, such as Bcl-2, Bcl-xL, and Mcl-1, and proapoptotic proteins, such as the BH3-only proteins Bid, Bad, Noxa, Puma, and Bim, as well as Bax, Bak, and Bok [20]. In response to death signals, monomeric Bax or Bak translocate from the cytosol to the outer mitochondrial membrane (OMM) where they form oligomers, causing mitochondrial permeabilization and release of cytochrome c and Smac/DIABLO. Released cytochrome c triggers caspase-9 activation and consequently caspase-3 activation. Smac/DIABLO impedes the function of inhibitor of apoptosis proteins, IAP and XIAP. Bcl-2 exerts its antiapoptotic function by binding to pro-apoptotic proteins, Bax and Bad, preventing their oligomerization and thus maintaining mitochondrial membrane integrity. BH3-only proteins compete with Bcl-2 for binding with apoptotic activators, thus promoting apoptosis [21].

The mitochondrial (intrinsic) pathway is activated during anoikis. Bid translocates to the OMM following loss of adhesion with identical kinetics with Bax, which is required for anoikis of mammary epithelial cells [22]. Bim is sequestered by dynein cytoskeletal complexes when cells are attached to ECM. Cell detachment induces release of Bim from these complexes and causes its translocation to the mitochondria, where it interacts with Bcl-xL, neutralizing its prosurvival function [23, 24]. Loss of cell adhesion also causes the accumulation of cytoplasmic Bim via inhibiting its proteasomal degradation initiated by its phosphorylation through the ERK and PI3K/Akt pathways. Activated Bim promotes Bax-Bak oligomerization within the OMM and induces intrinsic apoptosis pathway upon cell detachment [4, 24]. Bcl-2 modifying factor (BMF) is sequestered to myosin V motor complexes and, upon loss of cell attachment, is released. BMF then binds to antiapoptotic Bcl-2 to initiate anoikis in human intestinal epithelial cells [4, 25, 26]. Both Bim and BMF are associated with cytoskeletal structures and counteract the activity of antiapoptotic Bcl-2 when cell adhesion is disrupted. Therefore, suppression of Bim and BMF could confer anoikis resistance. In fact, overexpression of EGFR blocks both Bim expression and anoikis [27] and hypoxia decreases Bim and BMF expression and blocks anoikis in mammary epithelial cells [28].

## 4. EMT and Anoikis Resistance

During metastasis, epithelial cancer cells are released from their environment by breaking the basement membrane barrier. This process is involved in epithelial-mesenchymal transition (EMT). EMT is the biological process through which polarized epithelial cells undergo multiple biochemical changes, leading to a mesenchymal phenotype, such as enhanced migratory capacity, invasiveness, and resistance to apoptosis [29]. EMT is an essential process during development and is also induced during tissue repair and pathological processes, including inflammation and high-grade carcinomas in adults. EMT is characterized by loss of several epithelial proteins, including E-cadherin, *β*-catenin, and *γ*-catenin. It is also accompanied by increased expression of mesenchymal proteins, such as N-cadherin, vimentin, fibronectin, and smooth muscle actin. The significance of E-cadherin loss in metastasis has been shown in a variety of *in vitro* and *in vivo* models [30, 31] and loss of E-cadherin is the major hallmark of EMT. Loss of E-cadherin may be achieved through the upregulation of transcriptional repressors of E-cadherin, including E12/E47, Twist, and members of the Zeb and Snail protein families. Transforming growth factor-*β* (TGF-*β*), an inducer of EMT, upregulates these transcription factors, thereby promoting EMT and metastasis [32, 33].

EMT is not only a key event for epithelial-derived cells to acquire a motile and invasive phenotype but also an essential process for anoikis resistance [34]. EMT-promoting proteins are linked to anoikis resistance. Loss of E-cadherin induces anoikis resistance and promotes metastasis and N-cadherin expression also induces anoikis resistance [35]. Twist is the mediator of loss of E-cadherin-induced anoikis resistance [36, 37]. Conditional knockdown of E-cadherin and p53 in mammary epithelium induces mammary tumor initiation, metastasis, and anoikis resistance in the mouse model [37]. Moreover, the loss of E-cadherin induces Twist expression, indicating a feed-forward loop to maintain EMT [38]. Neurotrophic tyrosine kinase receptor B (TrkB) induces EMT, and knockdown of Twist blocks TrkB-induced EMT, anoikis resistance, and tumor growth. Moreover, Snail was induced by Twist, and silencing of Snail impairs EMT and anoikis resistance [39]. Twist is upregulated in several malignancies and promotes EMT, and Zeb and Snail are often overexpressed in metastasizing tumors [36, 40]. Knockdown of Zeb1 induces E-cadherin expression and inhibits cell growth in anchorage-independent conditions [41]. Zeb1, acting downstream of Twist and Snail, is also required for TrkB-induced EMT, anoikis resistance, and metastasis [42]. The E-cadherin-interacting protein, ankyrin-G, mediates anoikis regulatory signals, and binds with neurotrophin receptor-interacting MAGE homolog (NRAGE). NRAGE represses the *p14ARF* gene and suppresses anoikis [43]. Recently, Shin et al. [44] reported that activation of EKR2 but not EKR1 is necessary for Ras-induced EMT in MCF-10A cells that are transformed by oncogenic Ras. ERK2 activation results in the Fra1 upregulation, which in turn triggers the accumulation of Zeb1/2, thereby inducing EMT and increasing migration, invasion, and survival. In addition, TGF-*β* induces EMT via isoform switching of FGF receptors, causing the cells to be more sensitive to FGF-2, which activates the MEK-ERK pathway to regulate complex formation of Zeb1 with transcription corepressor CtBP1 [45].

**Figure 2:**
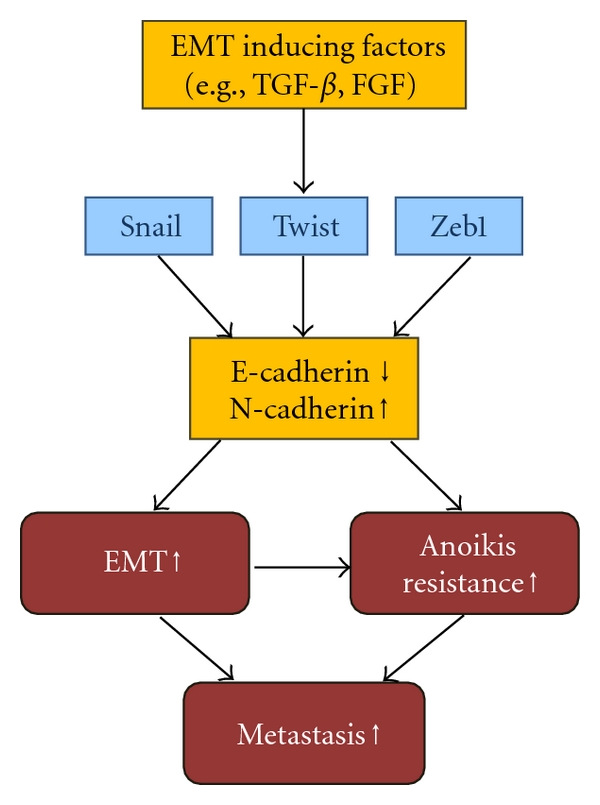
Schematic representation of EMT and anoikis resistance. EMT-inducing factors, such as TGF-*β* and FGF activate transcriptional factor, Twist, Snail and Zeb1. Activated these transcriptional factors repress E-cadherin (encoded CDH1 gene) expression and induce N-cadherin expression (Cadherin switch). Cadherin switch induces EMT and anoikis resistance, which are associated with tumor metastasis.

During the EMT process, E-cadherin expression is down-regulated whereas N-cadherin expression is up-regulated, referred to as a “cadherin switch” [46]. E/N-cadherin switch promotes cancer progression via TGF-*β*-induced EMT in extrahepatic cholangiocarcinoma [47]. N-cadherin expression appears to be more critical for tumor malignancy than E-cadherin. N-cadherin promotes cell motility and invasion via interactions with growth factor receptors such as FGF receptors and PDGF receptor. N-cadherin also promotes cell growth and survival by repressing apoptotic signals and numerous clinical studies have shown that aggressive human tumors express N-cadherin *in situ*, indicating a critical role for cadherin switch in human tumorigenesis [48]. Accordingly, both EMT and anoikis resistance are key processes for metastasis and they share common regulators, such as Twist, Snail, Zeb1, E-cadherin, and N-cadherin (Figure 2).

## 5. Growth Factor Receptors and Anoikis Resistance

Unregulated expression of growth factor receptors or components of their signaling pathways are associated with tumor malignancy due to their inhibition of cell death pathways and activation of cell survival pathways [49]. Abnormal regulation of growth factor receptors activates prosurvival signaling pathways, such as the PI3K/Akt, Ras/MAPK, NF-*κ*B, and Rho-GTPase pathways [1, 49], leading to metastasis by inhibiting anoikis. This can be achieved through autocrine signaling of growth factors, including fibroblast growth factor (FGF), hepatocyte growth factor (HGF), and platelet-derived growth factor (PDGF). In addition, overexpression of growth factor receptors, such as EGF receptor, TrkB receptor, and HGF receptor, can suppress anoikis.

ErbB family members are tightly linked to tumor progression and malignancies through the activation of many different signaling pathways for cell proliferation, survival, and migration [49]. Upon detachment from ECM, EGFR (ErbB1) is downregulated and Bim is upregulated, which is a critical mechanism for anoikis. Overexpression of EGF receptor maintains ERK activation in suspended cells and blocks anoikis via suppression of Bim expression in MCF-10A cells, indicating that growth factor receptor expression uncouples anoikis from integrin regulation [27]. Moreover, ErbB2 overexpression blocks Bim expression and anoikis, via upregulation of *α*5 integrin and activation of Src in ECM-detached cells [50]. Loss of ECM attachment downregulates EGF receptor and *β*1 integrin both at the protein and mRNA levels. However, ErbB2 overexpression rescues both EGF receptor and *β*1 integrin protein via ERK and Sprouty, which stabilizes EGF receptor in ECM-detached cells [51]. Cell detachment causes ATP deficiency due to the decreased glucose transport but this deficiency can be rescued by ErbB2 overexpression, which stabilizes EGF receptor expression and thus PI3K activation [52]. TGF-*α*, a ligand for EGF receptor, prevents anoikis of intestinal epithelial cells by reversing the loss of Src activity and Bcl-xL expression induced by cell detachment [53]. Anchorage-independent Ewing sarcoma cells suppress anoikis through a pathway involving E-cadherin cell-cell adhesion, which leads to ErbB4 activation of the PI3K/Akt pathway [54]. The PI3K/Akt pathway plays a critical role in cell survival and PTEN, one of the most frequently mutated tumor suppressors in human cancer, negatively regulates the PI3K/Akt pathway [55]. PTEN also plays an important role in the anoikis induction through negative regulation of FAK. Overexpression of PTEN induces anoikis via suppression of the phosphorylation of FAK and Akt in human bladder transitional carcinoma cells [56] and in U251 glioma cells [57]. Accordingly, loss of PTEN confers apoptotic resistance to cell rounding and matrix detachment in human mammary epithelial cells [58].

Insulin-like growth factor 1 (IGF-1) is a well-established cell survival factor that triggers Akt activation after loss of matrix contact [59]. IGF-1 receptor prevents anoikis in mouse embryo fibroblasts [60] and in the LNCaP human prostate epithelial cell line [59]. Disruption of IGF-1 receptor signaling decreased the number of circulating tumor cells in the blood of tumor-bearing mice and enhanced anoikis of LCC6 cells, a metastatic variant of MDA-MB-435 breast cancer cells [61]. Many studies have reported that PDGF receptor is also associated with metastasis of tumor [62–64]. PDGF receptor acts as the upstream tyrosine kinase for Src, a key contributor for anoikis resistance, in a human lung adenocarcinoma upon cell detachment [65]. The cooperation of autocrine PDGF-PDGFR signaling with oncogenic Ras strongly activates PI3K and is required for survival during EMT [66]. Transforming growth factor-*β* (TGF-*β*) and its receptor also play critical roles in tumor progression and metastasis. TGF-*β*1 coordinately and independently activates FAK and Akt kinase pathways through the early activation of SMAD3 and p38 MAPK, respectively, to confer an anoikis resistant phenotype to myofibroblasts [67]. In addition, in conjunction with EGF, TGF-*β*1 enhances migration, invasion, and anchorage-independent growth compared to that induced by EGF alone through the activation of MAPK and Akt [68]. Accordingly, the TGF-*β* inhibitor, LY2109761, suppresses metastasis of pancreatic cancer by inducing anoikis [69]. Conditional activation of FGF receptor causes anchorage-independent growth and EMT in a ribosomal S6 kinase-dependent manner in MCF-10A cells [70]. HGF and its receptor inhibits anoikis of pancreatic carcinoma cells through the PI3K pathway and of head and neck squamous carcinoma cells through activation of ERK and Akt signaling [71, 72]. Vascular endothelial growth factor A (VEGF-A) and its main signaling-receptor VEGFR2 (KDR) are expressed in primary ovarian tumors and autocrine VEGF-A/KDR loop protects ovarian carcinoma cells from anoikis [73].

## 6. Energy Metabolism, Autophagy, ROS, and Anoikis Resistance

Rapidly dividing tumor cells require rapid ATP generation, increased biosynthesis of biomolecules, and maintenance of an appropriate redox status to support cell division, despite the low oxygen (hypoxia) and nutrient levels within the tumor [74]. Tumor cells reprogram their metabolic pathways to meet these needs. The best-characterized metabolic phenotype in tumor cells is marked by the Warburg effect, which is a shift from ATP generation through oxidative phosphorylation to ATP generation through glycolysis, even under normal oxygen concentrations [75]. This aerobic glycolysis is regulated by PI3K/Akt [76, 77], hypoxia-inducible factor (HIF) [78–82], p53 [83–85], Myc [77, 86, 87], and AMP-activated protein kinase (AMPK)-liver kinase B1 (LKB1) [88–90] pathways. Alternatively, stress conditions, such as limited nutrients and hypoxia, activate autophagy to buffer metabolic stress during tumor growth [91].

An elegant model for the study of anoikis has been developed and provides insight into the mechanisms of detachment-induced apoptosis. Three dimensional culture of MCF-10A mammary epithelial cells form spheroid structures, termed acini, in which a layer of polarized epithelial cells surrounds a hollow lumen, resembling glandular epithelium *in vivo*. This lumen formation involves the clearance of central cells by selective anoikis of cells lacking ECM attachment [91]. When MCF-10A cells are detached from the ECM, glucose uptake is decreased, energy production is reduced [52]. Overexpression of ErbB2 has been linked to anoikis resistance. In line with this report, overexpression of ErbB2 rescues ATP deficiency by restoring glucose uptake through EGF receptor stabilization and PI3K activation, which is consistent with the studies that the PI3K/Akt pathway activates glucose uptake and protects cancer cells from starvation [52, 92]. Lack of glucose uptake in the detached cells blocks both glycolysis and the pentose phosphate pathway (PPP). In addition to macromolecular building blocks, PPP produces NADPH, a crucial cellular reducing agent, quenching the reactive oxygen species (ROS) produced during cell metabolism [74]. Accordingly, in the detached cells, ROS levels are increased and antioxidant treatment rescues low ATP levels by permitting fatty acid oxidation, leading to the filling of the luminal space in MCF-10A acini. An increase in oxidative damage blocks the consumption of fatty acids for energy production, and thus detached epithelial cells experience severe starvation and death. However, expression of the ErbB2 oncogene enables MCF-10A cells to overcome anoikis because there is enough ATP production with continuous glucose uptake.

Autophagy is a tightly regulated lysosomal self-eating process that is upregulated during cellular stress, such as deprivation of nutrients and growth factors. Autophagy produces nutrients and energy to enhance cell survival through the breakdown of cytosolic components. Basal levels of autophagy are required to maintain homeostasis by removing damaged proteins and organelles. Although autophagy is essential for cell survival, excessive autophagy results in programmed cell death. Autophagy is regulated by autophagy-related genes (ATGs), and the majority of pro-autophagic events converge on the mammalian target of rapamycin (mTOR) pathway [93]. Autophagy is known to suppress tumor formation by limiting chromosomal instability and promoting cellular senescence [94]. In contrast, protumorigenic functions for autophagy have been proposed and demonstrated. Autophagy is increased in cancer cells during many of the conditions directing metastasis, including hypoxia, metabolic stress, and cell detachment from ECM [93]. Silencing of autophagy regulators inhibits detachment-induced autophagy and enhances apoptosis, indicating that autophagy promotes epithelial cell survival during anoikis [91]. Recently, it was reported that oncogenic H-Ras expression induces autophagy upon cell detachment [95]. Interestingly, genetic deletion or RNAi-mediated knockdown of autophagy regulators decreases cell growth in soft agar and reduces glycolysis, indicating that an intact autophagy pathway is required for adhesion-independent transformation and facilitates glycolysis through oncogenic H-Ras [95]. These studies suggest that anoikis resistance is associated with tolerance to bioenergetic stress due to oncogene expression.

The balance between oxidation and reduction plays a critical role in the cellular signaling pathways involved in cell growth and metastasis. Elevated oxidative stress is more frequently observed in many solid tumors and carcinoma cell lines than in normal cells. ROS are now recognized as key second messengers during growth factor and cytokine stimulation to elicit prosurvival signals. Upon integrin engagement, ROS are produced via Rac-1, and elevated ROS induce prosurvival signaling through Src activation. Activated Src phosphorylates EGF receptor in a ligand-independent manner, which activates the ERK and Akt pathways, leading to Bim degradation and suppression of anoikis. Src activation plays a critical role in anoikis resistance. Src is transiently activated upon cell detachment, which delays anoikis in intestinal epithelial cells [96]. In addition, in metastatic prostate cancer cells, ROS are constitutively produced due to sustained activation of 5-lopoxigenase (5-LOX). These amplified and persistent redox signals activate Src in the absence of adhesion, thereby sustaining ligand-independent EGFR activation. This pathway degrades pro-apoptotic protein Bim, thereby promoting anoikis resistance [97]. Elevated expression of angiopoietin-like 4 (ANGPTL4) is widespread in tumors and its role in anoikis resistance has been recently reported [98]. ANGPTL4 binds to *β*1 and *β*5 integrins directly, which stimulates NADPH oxidase (NOX)-dependent production of O_2_^−^. A high ratio of O_2_^−^ : H_2_O_2_ activates Src, triggering the prosurvival PI3K/Akt and ERK pathways to confer anoikis resistance.

## 7. Membrane Microdomains and Anoikis Resistance

Another way to regulate anoikis is through modulation of membrane microdomains, including lipid rafts. This topic was recently reviewed [99]. There are two distinct types of lipid rafts: planar lipid rafts (noninvaginated rafts) and invaginated rafts known as caveolae. Caveolae are characterized by specific scaffolding proteins, called caveolins. Sphingolipids and cholesterol are the major lipid components of lipid rafts, and cholesterol is critical for the intact structure of lipid rafts. Because cell surface receptors, such as integrins and growth factor receptors, and some intracellular signaling molecules are enriched in lipid rafts, lipid rafts are regarded as the sites of assembly and initiation of signaling pathways [100, 101].

Caveolin-1, a structural protein for caveolae, acts as a scaffolding protein via its binding to signaling molecules such as EGF receptor, Src family members, MAPK, PKC, endothelial nitric oxide synthase (eNOS), and G-protein *α* subunits through its scaffolding domain [101]. This binding is known to negatively regulate these molecules. Initially, caveolin-1 was regarded as a tumor suppressor because it is downregulated in transformed cells and re-expression of caveolin-1 inhibits colony formation and induces apoptosis in transformed cells and breast cancer cells [102]. However, caveolin-1 levels are elevated in prostate cancer and lung adenocarcinomas, and the elevated caveolin-1 levels are associated with increased metastatic capacity and poor prognosis [103, 104], indicative of an oncogenic role for caveolin-1. Several studies have demonstrated that caveolin-1is associated with anchorage-independent growth. Caveolin-1 expression inhibits anoikis by inhibiting p53 activation and activating IGF receptor-mediated ERK and Akt signaling pathways upon cell detachment [105]. In human lung carcinoma H460 cells, caveolin-1 is downregulated during cell detachment through a mechanism involving ubiquitin-mediated proteasomal degradation. Interestingly, nitric oxide (NO) prevents downregulation of caveolin-1 by ubiquitination and this event suppresses anoikis [106]. Alternatively, increased levels of hydrogen peroxide in detached cells prevent caveolin-1 degradation and stabilize it, thereby inhibiting anoikis [107]. Overall, caveolin-1 appears to have dual functions depending on the cell type: as a tumor suppressor by inhibiting anchorage-independent cell growth and as a promoter of tumor metastasis by preventing anoikis.

Lipid rafts are associated with integrin signaling. Thus, modulation of lipid rafts is linked to adhesion-dependent cell survival. Cell detachment triggers internalization of lipid rafts, and inhibition of lipid raft internalization maintains Rac1 membrane targeting and downstream effector activation in suspended cells [108]. This study indicates that integrins regulate lipid raft localization, thereby controlling anchorage-dependent cell growth. In line with this notion, disruption of lipid rafts by cholesterol depletion results in FAK down-regulation, cell detachment, lipid raft internalization, and anoikis-like cell death in A431 human cervical cancer cells [109, 110]. Lipid raft disruption induces tyrosine phosphorylation of caveolin-1 through Src activation, which could be involved in lipid raft internalization. Caveolin-1 is tyrosine phosphorylated by Src and EGF-receptor signaling [99, 111], and this phosphorylation has been shown to be involved in caveolae internalization [112]. Under lipid raft disrupting stress, HIF-1*α* is induced via EGF receptor activation, which delays anoikis. This is consistent with the finding that knockdown of HIF-1*α* accelerates anoikis [113]. Aloe-emodin is an anthraquinone derivative that alters lipid rafts by decreasing sphingolipid and cholesterol in the lipid raft fraction. It inhibits tumor cell adhesion through disruption of the lipid raft-associated integrin signaling pathways, such as FAK recruitment to *β*1 integrin [114] and it sensitizes anoikis in gastric carcinoma cells and lung cancer H460 cells [115, 116]. Akt activation is important for cell survival and anoikis resistance [4]. Intact lipid rafts are critical for PI3K/Akt signaling because they facilitate Akt recruitment and activation upon phosphatidylinositol-3,4,5-triphosphate accumulation in the membrane [117]. Lipid rafts disruption by cholesterol depletion results in both lipid raft internalization and Akt inactivation even in the presence of EGF stimulation. Cholesterol repletion reverses these effects [109, 110], indicating that lipid raft localization in the plasma membrane is important for Akt activation. It is possible that metastatic tumor cells possess a mechanism to regulate lipid raft localization to escape anoikis, which remains to be investigated further.

## 8. Conclusion

Normal epithelial cells require adhesion to the appropriate ECM for survival and proliferation, and loss of this adhesion induces a type of cell death known as anoikis. Anoikis is important for normal development and tissue homeostasis because it prevents detached cells from reattaching to an inappropriate ECM and growing dysplastically. Anoikis resistance, or survival in the absence of attachment to ECM, is a prerequisite for the development of tumor metastases, the major cause of cancer mortality. Currently, anoikis dysregulation or resistance has evoked special attention in the cancer research fields because circulating tumor cells in the blood stream are resistant to anoikis, which allows the cancer cells to disseminate from the primary tumor site to a distinct lesion. Anoikis is regulated by many different signaling pathways depending on the cell type and expressed oncogenes. Both the intrinsic and extrinsic death pathways are employed in anoikis. Expression of oncogenes, such as ErbB family members, is enable cells to avoid anoikis by inhibiting apoptotic pathways. In this paper, we included current data on the mechanisms of anoikis in relation to alterations of energy metabolism, autophagy, ROS, and lipid rafts, which are emerging now as a major factor in the regulation of anoikis and remain to be explored further. A better understanding of the molecular mechanisms involved in anoikis resistance would assist in the development of anticancer drugs to eradicate circulating tumor cells and prevent tumor metastasis.
